# Supplementary material for: Emergent geometry and duality in the carbon nucleus
Source: Nat Commun. 2023 May 15;14:2777. doi: 10.1038/s41467-023-38391-y (PMC10185503; doi:10.1038/s41467-023-38391-y)
Supplement: Supplementary file 1 — Supplementary Information [file 41467_2023_38391_MOESM1_ESM.pdf]

Supplementary Information

Auxiliary-field lattice Monte Carlo

We start from a set of trial initial states $|\Phi_i\rangle$ with $i = 1, 2, \ldots, N_{\rm ch}$, for $N_{\rm ch}$ channels, and perform Euclidean time projection with the transfer matrix $M =: \exp(-\alpha_t H) :$, where the colons denote normal ordering and $\alpha_t = a_t/a$. The Hamiltonian is $H = T + V$, where the kinetic energy T is $\mathbf{p}^2/(2m_N)$ and the interaction V is defined in the main text in Eq. (1). The Euclidean projection amplitude at time step N_t is $Z_{kl}(N_t) = \langle\Phi_k|M^{N_t}|\Phi_l\rangle$. Each of the trial states $|\Phi_i\rangle$ is a Slater determinant of single-particle wave functions. For the cluster initial states, we consider an antisymmetrized product of alpha clusters with spatially distributed Gaussian wave packets, $\phi(\mathbf{r}) = \exp\left(-\mathbf{r}^2/2w^2\right)$, with $w = 1.6 - 1.8\,\text{fm}$ for the width of the wave packet. Each wave function is projected onto specific irreducible representations (*irrep*) of the cubic group [1, 2]. More details on such trial states can be found in Ref. [3]. The energies at Euclidean time step N_t are obtained from the eigenvalues of the adiabatic transfer matrix,

$$M^{(a)}_{qq'}(N_t) = \sum_{q''} Z^{-1}_{qq''}(N_t) Z_{q''q'}(N_t + 1). \tag{S1}$$

We convert the eigenvalues $\lambda_i(N_t)$ to energies using the relation $\exp(-\alpha_t E_i(N_t)) = \lambda_i(N_t)$. In the calculation, the two-body interactions among A nucleons are replaced by one-body interactions between single nucleons with auxiliary fields that are sampled by Monte Carlo methods [4].

Pinhole algorithm

We use the pinhole algorithm (PA) to determine a classical distribution for the nucleon positions, spins, and isospins. For this, we compute the amplitude

$$Z_{kl}(\mathbf{n}_1, \ldots, \mathbf{n}_A, N_t) = \langle\Phi_k|M^{N_t/2}\rho(\mathbf{n}_1, \ldots, \mathbf{n}_A)M^{N_t/2}|\Phi_l\rangle, \tag{S2}$$

with the A-body density operator $\rho(\mathbf{n}_1, \ldots, \mathbf{n}_A)$ constructed from the normal-ordered product of density operators $\rho(\mathbf{n}_i) = a^\dagger_i(\mathbf{n})a_i(\mathbf{n})$. The positions for A nucleons (or "pinholes") $\mathbf{n}_i$ are sampled stochastically. The finite size of the nucleons is accounted for by a random Gaussian smearing of the nucleon positions.

Nucleon density operators

The definition of the density operator $\tilde\rho(\mathbf{n})$ that appears in main text as Eq. (1) is given by [5]:

$$\tilde\rho(\mathbf{n}) = \sum_{i=1}^{A} \tilde a^\dagger_i(\mathbf{n})\tilde a_i(\mathbf{n}) + s_{\rm L} \sum_{|\mathbf{n}'-\mathbf{n}|=1} \sum_{i=1}^{A} \tilde a^\dagger_i(\mathbf{n}')\tilde a_i(\mathbf{n}'). \tag{S3}$$

Here $s_{\rm L}$ is the local smearing parameter, and the smeared annihilation operator is

$$\tilde a_i(\mathbf{n}) = a_i(\mathbf{n}) + s_{\rm NL} \sum_{|\mathbf{n}'-\mathbf{n}|=1} a_i(\mathbf{n}'), \tag{S4}$$

where $s_{\rm NL}$ is the non-local smearing parameter.

Euclidean time extrapolation

In NLEFT, energies and operator expectation values are computed using auxiliary-field lattice MC at finite Euclidean projection time. As an example, the low-energy spectrum is obtained from the transient energies $E_i(N_t) = -\log(\lambda_i(N_t))/\alpha_t$. Some extrapolation is then usually required to obtain the corresponding values at infinite projection time. For instance, we obtain energies at infinite projection time by fitting the lattice data using the *ansatz* [6, 7]

$$E_i(t) = \frac{E_i + \sum_{k=1}^{k_{\max}} (E_i + \Delta E_{i,k}) c_{i,k} e^{-\Delta E_{i,k} t}}{1 + \sum_{k=1}^{k_{\max}} c_{i,k} e^{-\Delta E_{i,k} t}}, \tag{S5}$$

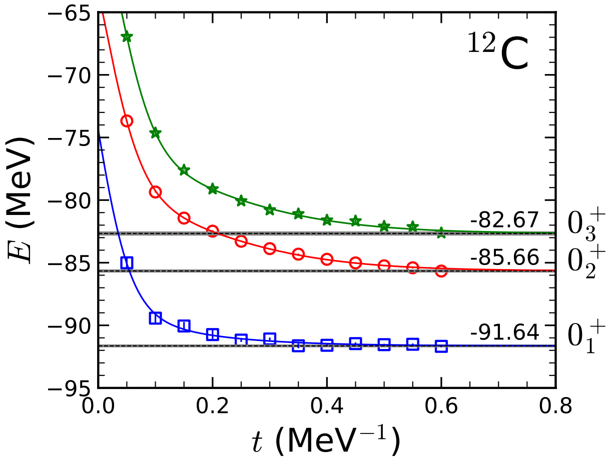

Supplementary Figure S1: Transient energies (open symbols) for the lowest three 0+ states versus Euclidean projection time using the SU(4) interaction. The data shown corresponds to a three-channel auxiliary field lattice MC calculation using alpha-cluster trial states and A_1^+ projection. The data are fitted and extrapolated using Eq. (S5). The widths of the horizontal lines reflect the extrapolation error.

where $E_i, \Delta E_{i,k}, c_{i,k}$ are fit parameters. The number of exponentials $k_{\rm max}$ should be kept as small as possible [6], in order to avoid fitting statistical fluctuations. For expectation value of operators of the type $\langle i|\hat{O}|i\rangle$, or transition operators $\langle i|\hat{O}|j\rangle$, we use the lowest order extrapolation formulas [7]

$$O_i(t) = \frac{O_i + O_{i,1}e^{-\Delta E_{i,1}t/2} + O_{i,2}e^{-\Delta E_{i,1}t}}{1 + c_{i,1}e^{\Delta E_{i,1}t}}, \qquad O_{ij}(t) = \frac{O_i + O_{i,1}e^{-\Delta E_{i,1}t/2} + O_{j,1}e^{-\Delta E_{j,1}t/2}}{1 + c_{i,1}e^{\Delta E_{i,1}t} + c_{j,1}e^{\Delta E_{j,1}t}}, \tag{S6}$$

with only one $\Delta E_{i,k}$ (or $\Delta E_{j,k}$). In Fig. **S1**, we show the extrapolation using Eq. (S5) for the lowest three 0^+ states of ^{12}C. With $k_{\rm max} = 2$, the convergence of the auxiliary-field MC data is well described.

SU(4) interaction parameters and systematic errors

In Table **S1**, we summarize the interaction parameters for the SU(4) interaction, together with the observables used to determine them. Our preferred set of parameters is denoted V1. It is then of interest to assess the systematic error associated with this choice of parameters. Since we are here considering an SU(4) symmetric interaction without a specific EFT power counting scheme, our strategy to estimate the systematic errors is to perform a number of fits with a different choice of the non-local smearing parameter $s_{\rm NL}$. The remaining three parameters $C_2, C_3, s_{\rm L}$ are again, in each case, fitted to the ground state energies of ^{4}He and ^{12}C, and to the electromagnetic radius of the ^{12}C ground state. While this procedure allows for a large number of possibilities, we have chosen two interactions denoted V2 and V3, for closer comparison.

The results of our systematic error analysis are given in Table **S1**. For simplicity, calculated energies and matrix elements are given at finite Euclidean projection time, as the additional computational effort required for a full extrapolation would not substantially alter the results nor the conclusions. Based on earlier experience, we take $t = 0.4\,{\rm MeV}^{-1}$ for ^{3}H and ^{12}C, and $t = 0.2\,{\rm MeV}^{-1}$ for ^{4}He. Such projection times should suffice for the purposes of the error analysis. From Table **S1**, we find that all three interactions give a similarly good description of the fitted observables. While the energy of the Hoyle state and the ^{4}He radius appear insensitive to the choice of interaction, the triton energy is influenced more. In particular, we observe that smaller values of $s_{\rm NL}$ give a more deeply bound ^{3}H. We find that such systematic errors for the ^{12}C spectrum do not exceed 2%, while for the alpha particle they are about $2\ldots3\%$. The radii of ^{4}He and ^{12}C are even less affected ($\leq 1\%$). For the bound states (0_1^+ and 2_1^+) and those near threshold ($0_2^+, 2_2^+, 3_1^-$), adding the 3NF has little influence. For the higher-lying states, the 3NF in general contributes more repulsion. Overall, the agreement with experiment is quite good.

In Fig. **S2**, we show the ^{12}C form factors obtained at finite Euclidean projection time $t = 0.4$ MeV^{-1} for the three interactions V1, V2, and V3, characterized by a different strength of the non-local smearing. As $s_{\rm L}$ is varied, the value of $s_{\rm NL}$ is adjusted so that ground state radius is kept constant. This results in the ground state form factor remaining almost the same for the three different interactions. There is also not a significant change to the overall charge density

Supplementary Table S1: Summary of interaction parameters $C_2, C_3, s_{\rm L}$, and $s_{\rm NL}$, and estimate of systematic uncertainty due to the choice of SU(4) interaction. The column labeled V1 denotes the preferred interaction used in this work, while V2 and V3 are used to quantify the systematic uncertainty. Observables shown are the ground state energies of ^{3}H, ^{4}He and ^{12}C, the energy of the Hoyle state, and the charge radii of ^{4}He and ^{12}C calculated by $r_c = \sqrt{r^2 + (0.84~{\rm fm})^2}$. The interaction parameters are fitted to $E_{^4{\rm He}}$, $E_{^{12}{\rm C}}$ and $r_{^{12}{\rm C}}$. As a measure of the resulting systematic error, we give $Q(2_1^+)$ along with the $E0$ and $E2$ transition matrix elements, for each interaction. For these, results at finite Euclidean time are shown for simplicity. The errors in the round brackets are purely statistical.

	V1	V2	V3
$s_{\rm NL}$	0.05	0.1	0.2
$s_{\rm L}$	0.08	0.071	0.06
C_2 [MeV^{-2}]	-2.15×10^{-5}	-1.11×10^{-5}	-3.47×10^{-6}
C_3 [MeV^{-5}]	6.17×10^{-12}	-5.92×10^{-13}	-1.46×10^{-12}
$E_{^4{\rm He}}$ [MeV]	$-28.1(1)$	$-28.3(1)$	$-27.3(1)$
$E_{^{12}{\rm C}}$ [MeV]	$-91.6(1)$	$-91.8(1)$	$-90.7(2)$
$r_{c,\,^{12}{\rm C}}$ [fm]	2.52(1)	2.55(1)	2.58(1)
$E_{\rm Hoyle}$ [MeV]	$-84.2(1)$	$-84.8(5)$	$-83.2(11)$
$E_{^3{\rm H}}$ [MeV]	$-10.1(1)$	$-8.1(1)$	$-5.1(1)$
$r_{c,\,^4{\rm He}}$ [fm]	1.63(1)	1.63(1)	1.64(1)
$Q(2_1^+)$ [$e\,$fm^2]	6.9(3)	7.2(4)	6.1(8)
$M(E0, 0_1^+ \to 0_2^+)$ [$e\,$fm^2]	4.3(3)	2.9(3)	5.9(7)
$B(E2, 2_1^+ \to 0_1^+)$ [e^2fm^4]	10.3(2)	10.7(3)	12.0(5)
$B(E2, 2_1^+ \to 0_2^+)$ [e^2fm^4]	1.8(1)	3.6(2)	4.1(5)

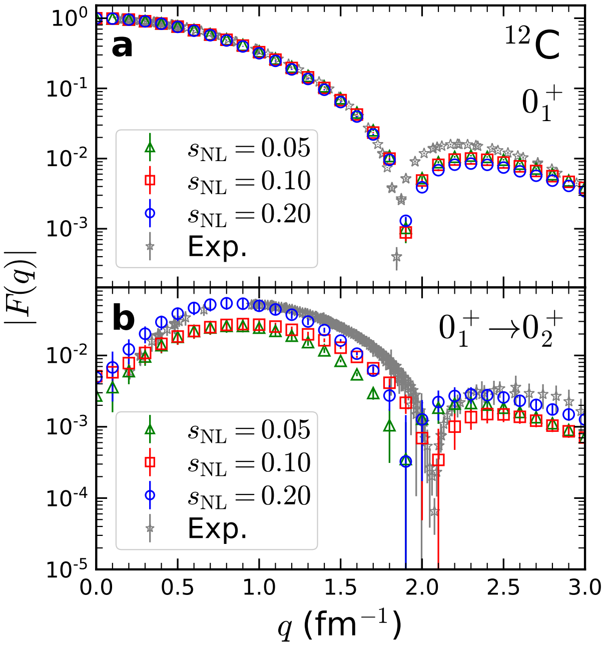

Supplementary Figure S2: Absolute value of the form factor for the ground state (**Panel a**) and the transition from the ground state to the Hoyle state (**Panel b**). Results are shown for Euclidean projection times $t = 0.4$ MeV^{-1} with three SU(4) interactions V1, V2 and V3 corresponding to $s_{\rm NL} = 0.1,\ 0.2,\ 0.05$, respectively. The comparison with experimental data is also shown [8, 9, 10, 5]. The error bars are one standard deviation uncertainties associated with stochastic errors.

of the Hoyle. However, the transition from the ground state to the Hoyle state is more sensitive to changes in $s_{\rm L}$. The shapes and positions of the maxima and minima of the transition form factor have some dependence on $s_{\rm L}$, and this

is also reflected in significant changes to the electromagnetic transitions between the Hoyle state and members of the ground state rotational band.

In a previous study of the ^{12}C spectrum using NLEFT [3], two different lattice spacings were used. The differences between the $a = 1.97$ fm results and $a = 1.64$ fm results can be explained by the significantly larger lattice errors at $a = 1.97$ fm. As shown in Ref. [11] for calculations of ^{8}Be, the lattice artifacts become significantly larger for $a > 1.7$ fm. This explains the choice of lattice spacing $a = 1.64$ fm for this analysis. Work currently in progress using a smaller lattice spacing of $a = 1.32$ fm provides confirmation that the lattice artifacts at $a = 1.64$ fm are less than 1 or 2 MeV in binding energy for the spectrum of ^{12}C. This is reflected in the excellent agreement with the observed binding energies.

N2LO chiral interaction parameters and results

The chiral interaction up to N2LO is defined as

$$V_{2\mathrm{N}} = V_{\mathrm{SU(4)}} + V_{\mathrm{OPE}} + V_{\mathrm{contact}}^{(Q/\Lambda_\chi)^0} + V_{\mathrm{contact}}^{(Q/\Lambda_\chi)^2} + V_{3N}, \tag{S7}$$

where Q is the low-energy scale (external momentum or pion mass), $\Lambda_\chi \simeq 700\,\mathrm{MeV}$ is the hard scale, V_{OPE} is the one-pion-exchange potential, and V_{3N} gives the leading three-nucleon force that appears at N2LO. Note that we have explicitly separated out the SU(4) symmetric pieces of the two- and three-nucleon contact terms that together with the one-pion exchange are treated non-perturbatively as leading order. All other operators appearing beyond LO are then treated in perturbation theory [12]. Note further that the two-pion exchange operators are expanded in powers of $(Q/\Lambda_\chi)^2$ and absorbed in the corresponding contact interactions [13]. Furthermore, the two-nucleon contact interactions are smeared with a Gaussian regulator to improve the description of the two- and other few-nucleon systems. In the S-waves, the corresponding cut-off is chosen as $\Lambda = 400$ MeV. The parameters of SU(4) and contact terms are determined by fitting to the phase shift of Nijmegen partial wave analysis (PWA93) [14], and are listed in Table **S2**. Galilean invariance is restored using the method in Ref. [15], and the coefficients are also listed in Table **S2**. The two low-energy constants of the three-nucleon interaction are determined then by fitting to the ground state energies of ^{4}He and ^{12}C, the corresponding LECs are also collected in Table **S2**.

Supplementary Table S2: Summary of N2LO chiral interaction parameters, all in lattice units.

	C_0	$C_{\mathrm{GIR},0}$	$C_{\mathrm{GIR},1}$	$C_{\mathrm{GIR},2}$	s_{NL}	s_{L}
SU(4)	−0.17395	−0.07001	0.01417	−0.00125	0.1	0.06
	C_0	$C_{\mathrm{GIR},0}$	$C_{\mathrm{GIR},1}$	$C_{\mathrm{GIR},2}$		
Q^0, $^1S_0$	0.44365	0.07410	−0.00980	−0.00128		
Q^0, $^3S_1$	−0.25149	−0.04505	0.01092	−0.00170		
Q^2, $^1S_0$	0.55249	0.02521	0.01665	−0.01042		
Q^2, $^3S_1$	−0.01090	1.15209	−0.64469	0.22634		
Q^2, $^1S_0^{0,1}$	0.03241	−0.03062	0.00780	−0.00135		
Q^2, $^3S_1^{0,1}$	0.02738	0.18172	−0.09556	0.03264		
Q^2, $^3S_1 - {}^3D_1$	−0.49342	0.09280	0.03828	−0.02687		
Q^2, $^1P_1$	0.96569	0.95481	−0.21826	0.02956		
Q^2, $^3P_0$	−0.19448	−0.07901	0.01729	−0.00206		
Q^2, $^3P_1$	0.92671	0.91735	−0.20962	0.02836		
Q^2, $^3P_2$	−0.04801	−0.03012	0.00730	−0.00114		
	c_D	c_E	s_{L}			
3N	−0.77527	0.67901	0.2			

The obtained phase shifts are given in Fig. **S3**, in comparison with Nijmegen partial wave analysis (PWA93) [14]. A good convergence is obtained at $(Q/\Lambda_\chi)^2$ order and a reasonably good accuracy can be achieved for the S-, P-, D-waves over the relative momentum range $0 < p_{\mathrm{rel}} < 200$ MeV. Note that due to the treatment of the two-pion exchange, these phase shifts are identical at NLO and N2LO.

Besides the Fig. **3** in the main text, we also show in Figs. **S4** and **S5** that the SU(4) interaction and the N2LO chiral interactions produce the same nuclear structures. In Fig. **S4** we compare SU(4) and N2LO chiral results for the 3_1^- and 4_1^+ states, both of which have good descriptions in terms of shell model states. In Fig. **S5** we show the SU(4) and N2LO chiral results for the radial charge densities for each of these states.

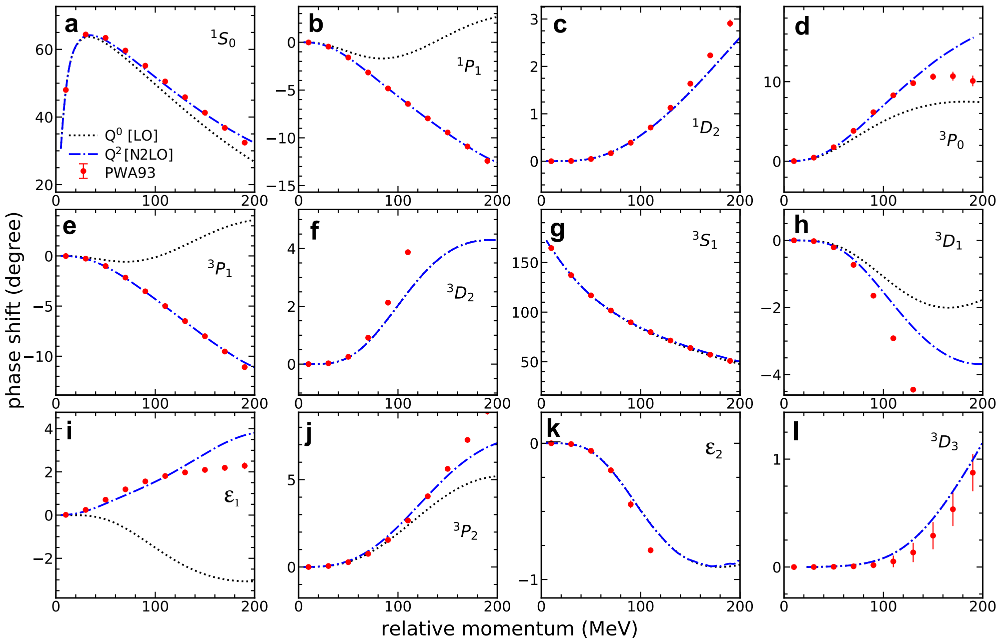

Supplementary Figure S3: Lattice phase shift up to N2LO, in comparison with the Nijmegen partial wave analysis (PWA93) [14]. **Panels a, b, c, d, e, f, g, h, i, j, k, l** show phase shifts and mixing angles for the $^1S_0$, $^1P_1$, $^1D_2$, $^3P_0$, $^3P_1$, $^3D_2$, $^3S_1$, $^3D_1$, ϵ_1, $^3P_2$, ϵ_2, $^3D_3$ channels respectively. The error bars are one standard deviation uncertainties associated with parameters determined from least squares fitting.

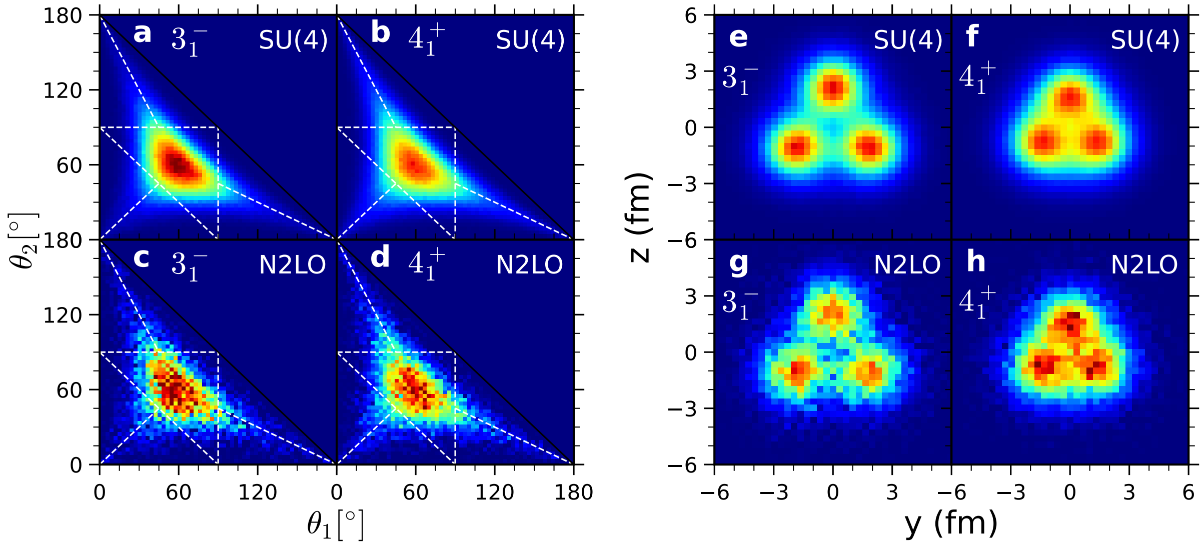

Supplementary Figure S4: Density distributions for the 3_1^- and 4_1^+ states. **Panels a, b, c, d:** Density distributions for the two inner angles of the triangle formed by the three alpha-clusters. **Panels a** and **c** are for the 3_1^- state using the SU(4) and N2LO interactions. **Panels b** and **d** are for the 4_1^+ using the SU(4) and N2LO interactions. **Panels e, f, g, h:** Two-dimensional projection of the nuclear density. **Panels e** and **g** are for the 3_1^- state using the SU(4) and N2LO interactions. **Panels f** and **h** are for the 4_1^+ state using the SU(4) and N2LO interactions.

Electromagnetic observables

Details on the calculation of electromagnetic observables can be found in the literature [16]. Here, we only give the final results and discuss issues directly relevant to the lattice calculation. The quadrupole moment Q for a given state

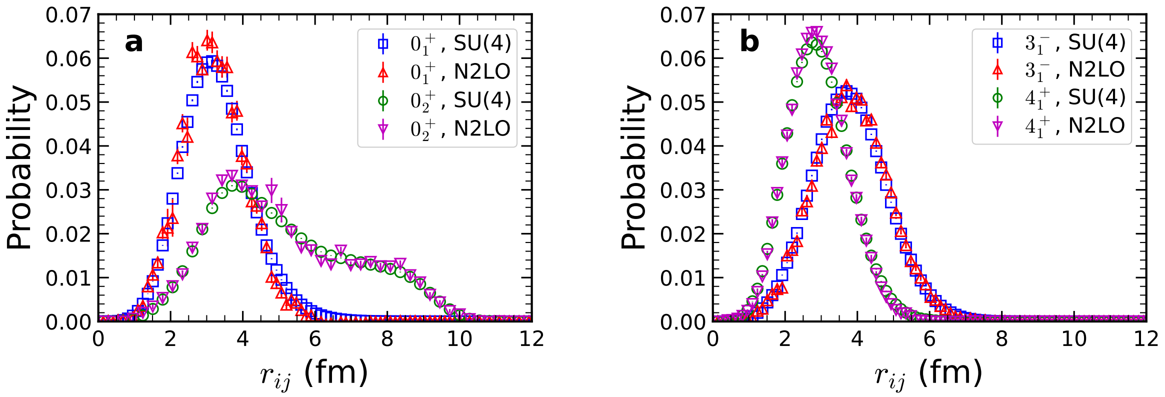

Supplementary Figure S5: Probability distribution for the distance between two alpha particles for the low-lying states of ^{12}C. We compare results using the SU(4) interaction and chiral interaction at N2LO. **Panel a** shows the 0_1^+ and 0_2^+ states. **Panel b** shows the 3_1^- and 4_1^+ states. The error bars are one standard deviation uncertainties associated with stochastic errors.

with angular momentum I and projection M along the z-axis is defined as

$$Q = \langle I, M = I|\hat{Q}|I, M = I\rangle, \tag{S8}$$

where the quadrupole moment operator is given by

$$e\hat{Q} = \int \rho_c(\mathbf{r})r^2(3\cos^2\theta - 1)d\tau, \tag{S9}$$

with e the electric charge, ρ_c the charge density distribution and $d\tau$ is the integration measure for the whole space. The reduced transition probability $B(E2)$ is defined as

$$B(E2, I_1 \to I_2) = \sum_{\mu, M_2} |\langle I_2 M_2|\mathcal{M}(E2,\mu)|I_1 M_1\rangle|^2. \tag{S10}$$

The electric quadrupole operator for different μ components is given by

$$\mathcal{M}(E2,\mu) = \int \rho_c(\mathbf{r})r^2 Y_{2\mu}(\theta,\varphi)d\tau, \tag{S11}$$

with $Y_{2\mu}(\theta,\varphi)$ the pertinent spherical harmonics.

In the above expressions, the coordinate $\mathbf{r}$ for each nucleon is defined in the CM frame of the nucleus. For the quadrupole moment and the $B(E2)$ strength, the operator to be evaluated is r^2 (or $x^2, \ldots, xy, \ldots$, depending on the choice of μ), as for the RMS radius. We evaluate expressions such as

$$\begin{aligned}\langle r_p^2\rangle &= \int \prod_{i=1}^{A} d\mathbf{r}_i |\Psi(\mathbf{r}_1,\mathbf{r}_2,\ldots,\mathbf{r}_A)|^2 \left[\sum_{j=1}^{Z}\hat{\mathbf{r}}_j - \frac{1}{A}\int \prod_{l=1}^{A} d\mathbf{r}'_l |\Psi(\mathbf{r}'_1,\mathbf{r}'_2,\ldots,\mathbf{r}'_A)|^2 \sum_{k=1}^{A}\hat{\mathbf{r}}'_k\right]^2 \\ &= \frac{1}{A}\int \prod_{i=1}^{A} d\mathbf{r}_i d\mathbf{r}'_i |\Psi(\mathbf{r}_1,\mathbf{r}_2,\ldots,\mathbf{r}_A)|^2 \left[\left(\sum_{j=1}^{Z}\hat{\mathbf{r}}_j - \sum_{k=1}^{A}\hat{\mathbf{r}}'_k\right)\right]^2 |\Psi(\mathbf{r}'_1,\mathbf{r}'_2,\ldots,\mathbf{r}'_A)|^2 \\ &- \frac{Z}{2A^2}\int \prod_{i=1}^{A} d\mathbf{r}_i d\mathbf{r}'_i |\Psi(\mathbf{r}_1,\mathbf{r}_2,\ldots,\mathbf{r}_A)|^2 \left[\left(\sum_{j=1}^{A}\hat{\mathbf{r}}_j - \sum_{k=1}^{A}\hat{\mathbf{r}}'_k\right)\right]^2 |\Psi(\mathbf{r}'_1,\mathbf{r}'_2,\ldots,\mathbf{r}'_A)|^2,\end{aligned} \tag{S12}$$

where we consider the proton density with Z the number of protons and A the number of nucleons.

For the quadrupole moment and the $B(E2)$ matrix element have a similar form. For example, when $\mu = 0$ the operator is proportional to

$$Q \sim 3z^2 - r^2, \tag{S13}$$

which can be treated along the lines of Eq. (S12).

Density projection of lattice states

In the NLEFT simulation for a given state with good angular momentum, the wave function is projected onto a given *irrep* of the cubic group. For example, the case of $J^\pi = 0^+$ corresponds to the A_1^+ *irrep* [1], which entails an equal superposition of all possible rotations of the wave function. In other words, for 0^+ states there is no preference in the angular distribution and the density distribution should be spherical. However, one may inquire as to the intrinsic shape of a ^{12}C nucleus, without such a superposition of all possible spatial rotations. Here, we adopt the following strategy: With the PA we can obtain the superposition of a large number of coordinates r_i ($i = 1, \ldots, A$) for A nucleons in all possible rotations. For the ground state, we already have the (model-independent) information from Fig. **2** (a) that an equilateral triangular configuration is preferred. Hence, for each configuration r_i we calculate its principal axis and align it in such a way that the shortest axis coincides with the x-axis. In this way, the three alpha clusters will be located on the $y - z$ plane. This can be achieved by calculating the matrix

$$R = \sum_{i=1}^{A} \begin{pmatrix} x_i^2 & x_i y_i & x_i z_i \\ y_i x_i & y_i^2 & y_i z_i \\ z_i x_i & z_i y_i & z_i^2 \end{pmatrix} \tag{S14}$$

and solving the eigenvalue problem

$$Rv = \lambda v. \tag{S15}$$

The resulting three eigenvalues correspond to the length along the longest, shortest, and intermediate principal axes, respectively. The eigenvectors can be used to rotate the original distribution so that the longest, shortest, and intermediate principal axes coinciding with the coordinate axes. For example, if we want to align the shortest axis with the x-axis, the intermediate axis with the y-axis, and the longest axis with the z-axis, we let $v_{\rm min}, v_{\rm mid}$, and $v_{\rm max}$ be the corresponding eigenvectors, and the rotation matrix can be constructed as

$$O = (v_{\rm min}, v_{\rm mid}, v_{\rm max}). \tag{S16}$$

Then one can rotate all particles $i = 1, \ldots, A$ from the old coordinates to the new ones as

$$(x, y, z)_{\rm new} = (x, y, z)_{\rm old} \times O. \tag{S17}$$

For nuclear states that we have already identified as having an equilateral shape, we rotate the configuration along the x-axis so that one of the alpha clusters is positioned on the positive z axis. We then symmetrize with respect to $0°$, $120°$, and $240°$ rotations. For nuclear states that we have already identified as having an obtuse isosceles shape, we identify the z axis as the direction with the longest RMS deviation of the nucleon positions relative to the CM. We then rotate the density configurations so that the alpha cluster closest to the CM lies on the positive y axis.

In Fig. **S6**, we show the density distribution for the two inner angles of the triangle in the top panel and the two-dimensional projection of the nuclear density in the middle panel for the states $1_1^-, 4_1^+, 1_2^+, 2_3^+, 4_2^-$ obtained using shell model initial states. The color scales are same as in Fig. **2**. The corresponding orbitals are shown in the lower panel of Fig. **S6**. The red circles are for protons and blue circles for neutrons, with solid ones for particles and hollow ones for holes. For those configurations not showing neutrons explicitly, the neutrons are occupied in the lowest possible levels (that is, fully $s_{1/2}$ and $1p_{3/2}$). They all show a similar equilateral triangle structure as the ground state, but in some cases with a less of a pronounced alpha cluster structure. It is also interesting to see that among those selected states, the alpha cluster structure is less pronounced when the particle excitation is in $s - d$ shell rather than the $1p_{1/2}$ level. Furthermore, the separation between the clusters is larger when the particle excitation reaches the $1d_{5/2}$ level.

For completeness, the density distributions for the other six states calculated in this work are shown in Fig. **S7** and are ordered by excitation energies. The color scales are the same as in Fig. **2**. The 4_2^+ appears to be a rotational excitation of the Hoyle state, while the 1_1^+ is similar to the Hoyle state but with a smaller angle and more compact structure. The $2_1^-, 1_1^+, 4_2^+$, and 4_1^- are obtained with cluster wave functions, while 2_2^- is obtained with shell model wave function $2s_{1/2} \otimes 1p_{3/2}$. The 0_4^+ is obtained using a shell model state with two-particle and two-holes in the $1p_{1/2} \otimes 1p_{3/2}$ orbitals (see the schematic plot in Fig. **S6**).

We should emphasize that the intrinsic shapes we are showing are not the result of initial state bias. This can be seen clearly in Fig. **S8** for the ground state of ^{12}C. In the upper panel (a)-(d), the initial state is a shell model wave function with harmonic oscillator orbitals. In the lower panel (e)-(h), the initial state is a cluster wave function. We see that the density distributions from very different initial wave functions begin to look similar as the projection time increases. This independence with respect to the choice of initial state was also demonstrated in previous work [17, 18, 3]. At Euclidean projection time $t = 0.2\,\text{MeV}^{-1}$ each of the distributions in the upper and lower panels look similar to the one shown for the converged ground state in Fig. **2**, obtained at larger values of t. At projection time $t = 0$ MeV^{-1},

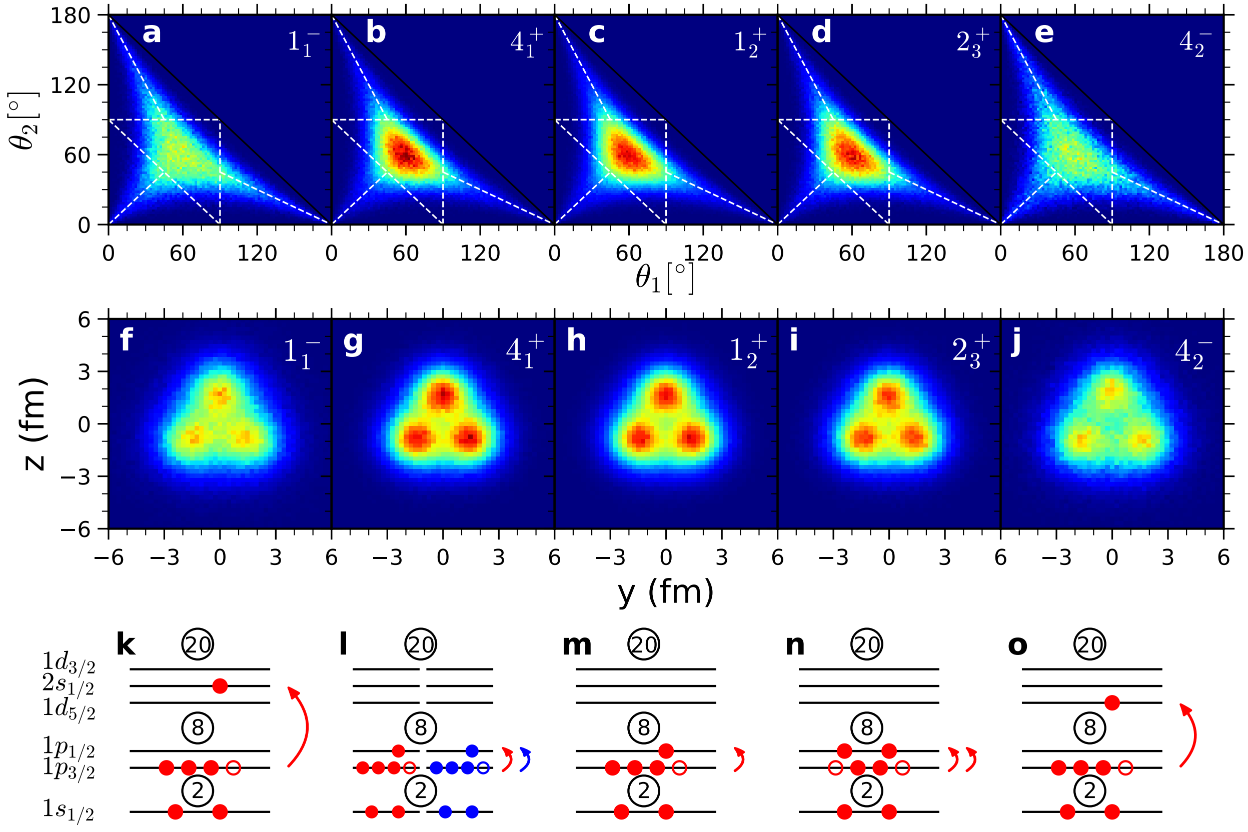

Supplementary Figure S6: Densities and configurations for the 1_1^-, 4_1^+, 1_2^+, 2_3^+, 4_2^- states. **Panels a, b, c, d, e** show the density distribution for the two inner angles of the triangle formed by the three alpha clusters for the 1_1^-, 4_1^+, 1_2^+, 2_3^+, 4_2^- states respectively using the SU(4) interaction. **Panels f, g, h, i, j** shows the two-dimensional projection of the nuclear density for the 1_1^-, 4_1^+, 1_2^+, 2_3^+, 4_2^- states respectively. **Panels k, l, m, n, o** sketch the orbitals for the shell model initial states used for the 1_1^-, 4_1^+, 1_2^+, 2_3^+, 4_2^- states respectively.

the intrinsic density for the shell model initial wave function is quite different from the converged ground state. In particular, there is a higher probability at the center and less probability to be restricted to the $y-z$ plane, and it is therefore more spherical.

Model-independent probes of cluster geometry

To more clearly assess the angular distribution of the various states, we show in Fig. S9 the probability of θ_3 when $\theta_1 = \theta_2$, which corresponds to the diagonal line along $\theta_1 = \theta_2 \in [0^\circ, 90^\circ]$ in Fig. 2. For the ground state, we find a clear peak around 60° as discussed above, but there is also a non-vanishing probability for an obtuse triangle ($\theta_3 > 90^\circ$). For the Hoyle state, there is little probability for an acute triangle, with a peak probability around 170°. The probability to form an exactly linear chain appears to be strongly suppressed ($\theta_3 \in [176^\circ, 180^\circ]$ with 4° resolution). This supports the notion that the intrinsic shape of Hoyle state is a large-angle obtuse triangle, but not exactly a linear chain. The lowest two 2^+ states show the same intrinsic shapes as the corresponding 0^+ states, which gives credit to the assertion made e.g. in Ref. [18] that the 2_2^+ state is a rotational excitation of the Hoyle state. We also note that the lowest negative-parity state, the 3_1^-, shows a distribution similar to the ground state. The angular distribution of the 0_3^+ state is very similar to the Hoyle state, but with a reduced probability for an exactly isosceles triangles with $\theta_1 = \theta_2$. This could be interpreted as a signal of a small-amplitude vibrational excitation.

Further model-independent information can be gained from Fig. S10, where the probability distributions for the distance between two alpha particles are shown, for the six lowest states in ^{12}C. The distance between two alpha particles is state-dependent, for the ground state it peaks at ~ 3 fm and for the Hoyle state near ~ 4 fm, as can also seen from the corresponding density distributions in Fig. 2. For the Hoyle state, one observes an extended tail, which

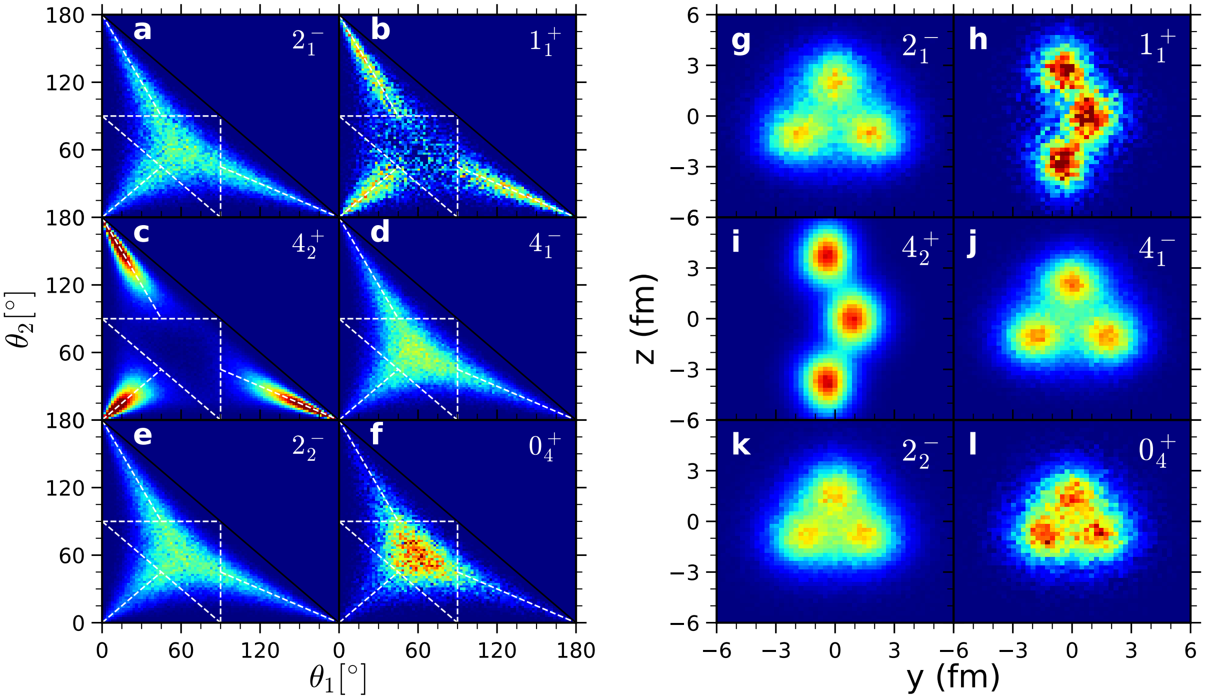

Supplementary Figure S7: Densities for the 2_1^-, 1_1^+, 4_2^+, 4_1^-, 2_2^-, 0_4^+ states. **Panels a, b, c, d, e, f** show the density distribution for the two inner angles of the triangle formed by the three alpha clusters for the 2_1^-, 1_1^+, 4_2^+, 4_1^-, 2_2^-, 0_4^+ states respectively using the SU(4) interaction. **Panels g, h, i, j, k, l** show the two-dimensional projection of the nuclear density for the 2_1^-, 1_1^+, 4_2^+, 4_1^-, 2_2^-, 0_4^+ states respectively.

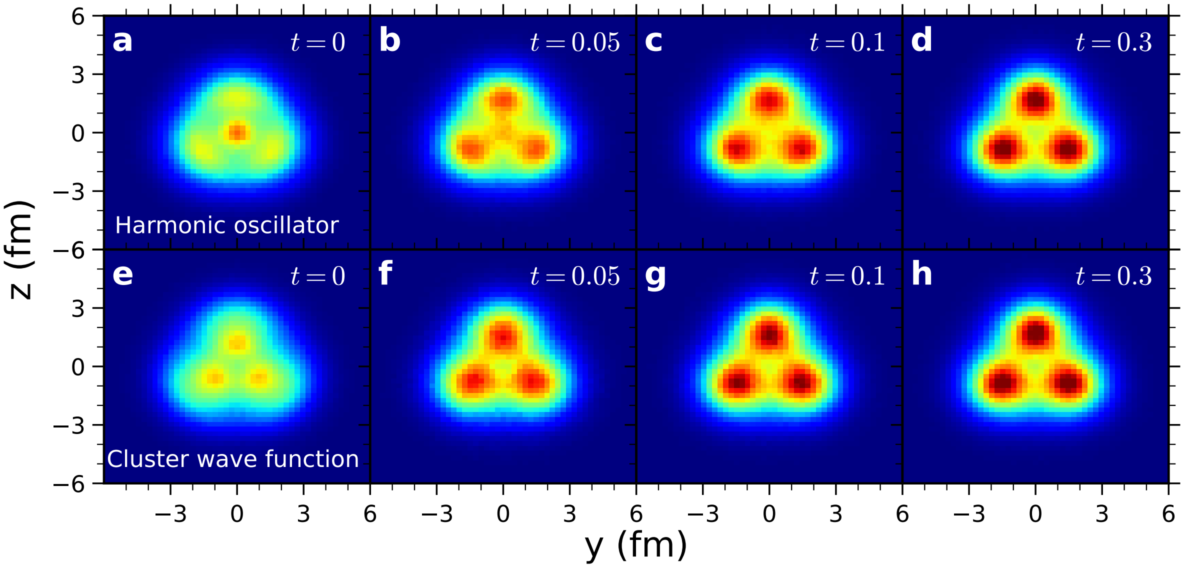

Supplementary Figure S8: Nuclear density for the ground state of ^{12}C versus projection time. We show results obtained using the SU(4) interaction with Euclidean projection time ranging from $t = 0$ to 0.2 in units in MeV^{-1}. For **Panels a, b, c, d**, the initial state is a shell model wave function composed of harmonic oscillator orbitals. For **Panels e, f, g, h**, the initial state is a cluster wave function.

reflects the distance between the two alpha particles associated with the longest side of the obtuse triangle. Again, we

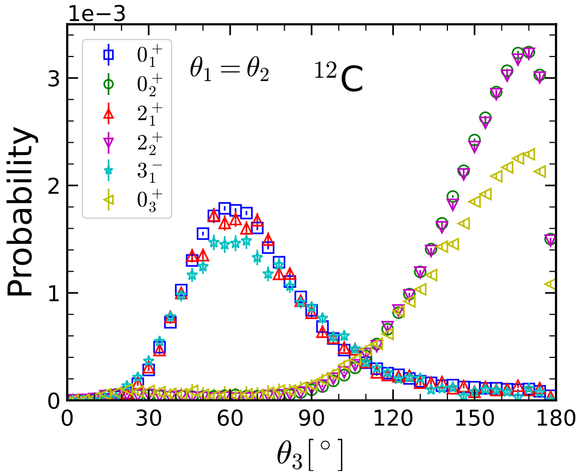

Supplementary Figure S9: Probability distribution versus angle. We show the probability distribution for the third angle of the triangle formed by the three alpha clusters of ^{12}C when the other two angles are equal ($\theta_1 = \theta_2$). Calculations are performed using the SU(4) interaction. This coincides with the diagonal line along $\theta_1 = \theta_2$ in Fig. 2. The error bars are one standard deviation uncertainties associated with stochastic errors.

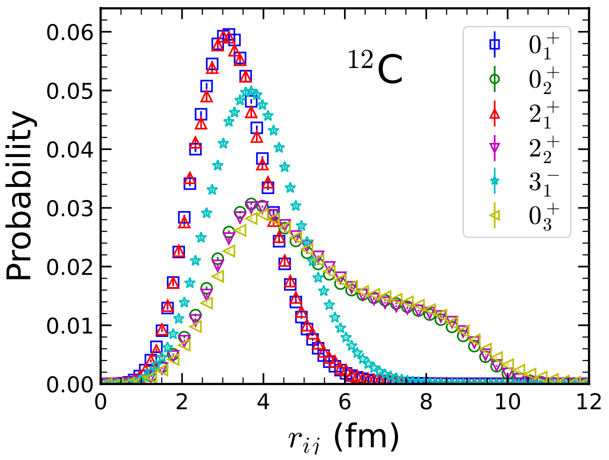

Supplementary Figure S10: Probability distribution versus distance. Probability distribution for the distance between two alpha particles, for the low-lying states of ^{12}C. Calculations are performed using the SU(4) interaction. The error bars are one standard deviation uncertainties associated with stochastic errors.

note the stunning similarity in this distribution for the $0_1^+, 2_1^+$ and $0_2^+, 2_2^+$ states, reinforcing the notion of the 2^+ states being rotational excitations of the corresponding 0^+ states. Note also that the 3_1^- state is somewhat broader than the ground state, but by far less extended than the Hoyle state. The similarity of 0_3^+ and Hoyle state, but with a slightly larger separation distance distribution, again supports the notion of a small amplitude vibrational excitation.

Electromagnetic density distributions

Here, we display the electromagnetic density distribution not shown in the main text. In Fig. **S11** the proton radial density distribution of the 0_1^+, 0_2^+ states is displayed, and the transition of $0_1^+ \to 0_2^+$ in comparison with the available experimental data [19, 5]. The density distribution of the ground state is nicely reproduced. The slight decrease in the center at $r = 0$ fm reflects the fact that the three alpha clusters are equally far away from the center. For the Hoyle state, the density distribution shown in Fig. **S11** (a) has no such decrease. This is also consistent from the shape information, that one alpha cluster is closely located near the center. The decrease of the density distribution as r increases has a visible slow down from $r = 2$ fm to $r = 6$ fm, and this corresponds to the separation between the two alpha clusters forming the longest side of the obtuse triangle. The pattern of the transition density $0_1^+ \to 0_2^+$ is also reproduced, with

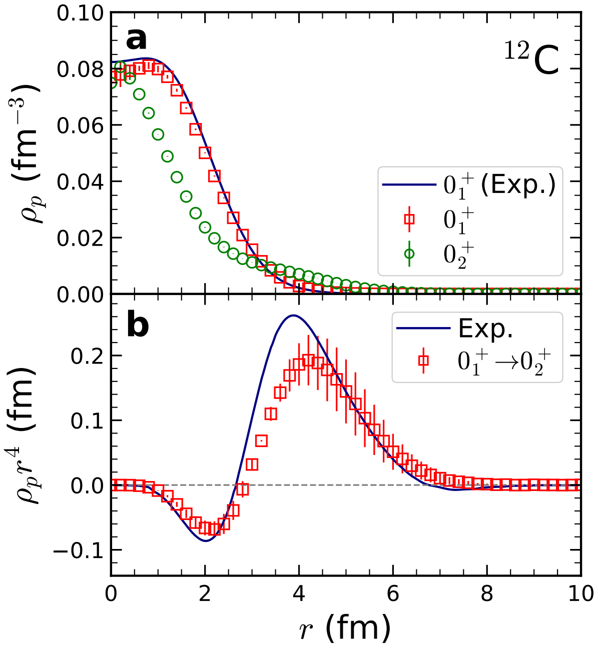

Supplementary Figure S11: Proton densities and transition amplitude versus radius. **Panel a** shows the proton densities of ^{12}C calculated by NLEFT in comparison with experiment [19, 5] for the ground state and the Hoyle state. **Panel b** shows the transition from the ground state to the Hoyle state. Calculations are performed using the SU(4) interaction. The error bars are one standard deviation uncertainties associated with stochastic errors.

a minimum near $r = 2$ fm, which is the approximate distance from the center to the α clusters in the ground state. The maximum occurs near $r = 4$ fm, which is the approximate distance from the center of the Hoyle state to the two α clusters furthest from the center.

Supplementary References

[1] R. C. Johnson, "Angular Momentum on a Lattice," Phys. Lett. B **114**, 147-151 (1982). doi:10.1016/0370-2693(82)90134-4

[2] B. N. Lu, T. A. Lähde, D. Lee and U.-G. Meißner, "Breaking and restoration of rotational symmetry on the lattice for bound state multiplets," Phys. Rev. D **90**, no.3, 034507 (2014). doi:10.1103/PhysRevD.90.034507

[3] S. Shen, T. A. Lähde, D. Lee and U.-G. Meißner, "Wigner SU(4) symmetry, clustering, and the spectrum of ^{12}C," Eur. Phys. J. A **57**, no.9, 276 (2021). doi:10.1140/epja/s10050-021-00586-6

[4] T. A. Lähde and U.-G. Meißner, "Nuclear Lattice Effective Field Theory: An introduction," Lect. Notes Phys. **957**, 1-396 (2019). doi:10.1007/978-3-030-14189-9

[5] S. Elhatisari, E. Epelbaum, H. Krebs, T. A. Lähde, D. Lee, N. Li, B. N. Lu, U.-G. Meißner and G. Rupak, "Ab initio Calculations of the Isotopic Dependence of Nuclear Clustering," Phys. Rev. Lett. **119**, no.22, 222505 (2017). doi:10.1103/PhysRevLett.119.222505

[6] T. A. Lähde, E. Epelbaum, H. Krebs, D. Lee, U.-G. Meißner and G. Rupak, "Uncertainties of Euclidean Time Extrapolation in Lattice Effective Field Theory," J. Phys. G **42**, no.3, 034012 (2015). doi:10.1088/0954-3899/42/3/034012

[7] R. He, N. Li, B. N. Lu and D. Lee, "Superfluid Condensate Fraction and Pairing Wave Function of the Unitary Fermi Gas," Phys. Rev. A **101** (2020) no.6, 063615. doi:10.1103/PhysRevA.101.063615

[8] P. Strehl, "Study of $0^+ - 0+$ transitions in 12C, 24Mg, 28Si, 32S and 40Ca by inelastic electron scattering", Z. Phys. **234** (1970) 416.

[9] H. Crannell, X. Jiang, J. T. O'Brien, D. I. Sober and E. Offermann, "Measurement of the pair transition width for the decay of the 7.654 MeV, 0 + state in 12 C," Nucl. Phys. A **758**, 399-402 (2005). doi:10.1016/j.nuclphysa.2005.05.072

[10] I. Sick and J. S. Mccarthy, "Elastic electron scattering from C-12 and O-16," Nucl. Phys. A **150**, 631-654 (1970). doi:10.1016/0375-9474(70)90423-9

[11] B. N. Lu, T. A. Lähde, D. Lee and U.-G. Meißner, "Precise determination of lattice phase shifts and mixing angles," Phys. Lett. B **760**, 309-313 (2016). doi:10.1016/j.physletb.2016.06.081

[12] B. N. Lu, N. Li, S. Elhatisari, Y. Z. Ma, D. Lee and U.-G. Meißner, "Perturbative Quantum Monte Carlo Method for Nuclear Physics," Phys. Rev. Lett. **128** (2022) no. 24, 242501. doi:10.1103/PhysRevLett.128.242501

[13] E. Epelbaum, H. Krebs, D. Lee and U.-G. Meißner, "Ground state energy of dilute neutron matter at next-to-leading order in lattice chiral effective field theory," Eur. Phys. J. A **40** (2009), 199-213. doi:10.1140/epja/i2009-10755-0

[14] V. G. J. Stoks, R. A. M. Klomp, M. C. M. Rentmeester, and J. J. de Swart, "Partial-wave analysis of all nucleon-nucleon scattering data below 350 MeV", Phys. Rev. C **48**, 792 (2020)

[15] N. Li, S. Elhatisari, E. Epelbaum, D. Lee, B. Lu, and U.-G. Meißner, "Galilean invariance restoration on the lattice", Phys. Rev. C **99**, 064001 (2019).

[16] A. Bohr and B. R. Mottelson, *Nuclear Structure*, World Scientific, Singapore (1969).

[17] E. Epelbaum, H. Krebs, D. Lee and U.-G. Meißner, "Ab initio calculation of the Hoyle state," Phys. Rev. Lett. **106**, 192501 (2011). doi:10.1103/PhysRevLett.106.192501

[18] E. Epelbaum, H. Krebs, T. A. Lähde, D. Lee and U.-G. Meißner, "Structure and rotations of the Hoyle state," Phys. Rev. Lett. **109**, 252501 (2012). doi:10.1103/PhysRevLett.109.252501

[19] Nuclei Charge Density Archive,
http://discovery.phys.virginia.edu/research/groups/ncd/index.html
